# Postural orthostatic tachycardia syndrome is associated with platelet storage pool deficiency

**DOI:** 10.1097/MD.0000000000004849

**Published:** 2016-09-16

**Authors:** William T. Gunning, Beverly L. Karabin, Thomas M. Blomquist, Blair P. Grubb

**Affiliations:** a Department of Pathology; b Department of Medicine, University of Toledo Medical Center, Toledo, OH.

**Keywords:** platelets, POTS, serotonin, storage pool deficiency, tachycardia

## Abstract

Mechanisms have been postulated to explain postural orthostatic tachycardia syndrome (POTS), however, the etiology of this often debilitating disorder remains unknown. We conducted a retrospective case–control study of 181 POTS patients who exhibited/reported bleeding symptoms for a specific platelet (PL) dysfunction disorder, delta granule storage pool deficiency (δ-SPD).

Patients were included only if results of blood tests for δ-SPD were available. Electron microscopy was utilized to diagnose δ-SPD. An ELISA assay was used to determine serotonin (5HT) concentration in PLs and medical record review was employed to collect patients’ clinical symptoms.

The most common bleeding symptom was easy bruising (71%) but frequent nose bleeds, heavy menstrual bleeding, and a family history of bleeding were also commonly reported. Of the patients studied, 81% were diagnosed with δ-SPD. Our investigation of 5HT concentration extracted from PLs revealed significantly lower levels of 5HT in POTS patients when compared to that of control subjects. Our data suggest that patients with POTS have significant comorbidities including bleeding symptoms and/or family bleeding histories, and have diminished PL 5HT levels supporting the hypothesis that POTS is a low 5HT level disorder. While we describe a significant relationship with POTS and δ-SPD, this finding does not constitute an etiology for POTS.

Our results establish an additional comorbidity frequently seen in POTS that could explain a number of disparate symptoms often affecting the severity of POTS.

## 1 Introduction

Postural orthostatic tachycardia syndrome (POTS) is defined clinically as the presence of symptoms of orthostatic intolerance associated with increased heart rate of 30 beats per minute (BPM) from the basal rate or a rate that exceeds 120 BPM that occurs within the first 10 minutes of standing or upright tilt. It is not associated with chronic debilitating conditions such as prolonged bed rest or the use of medications known to diminish vascular or autonomic tone.^[1]^ The syndrome is an abnormal physiological state that is commonly due to an inability of the peripheral vasculature to maintain adequate resistance in the face of orthostatic stress resulting in excessive pooling of blood in the more dependent areas of the body.^[2–4]^ This functional decline in circulatory volume will elicit a compensatory increase in heart rate and myocardial contractility. While compensatory in mild cases, this mechanism is unable to fully compensate in more severe cases, resulting in a reduction in effective circulation and varying degrees of cerebral hypoperfusion. A reduction in arterial blood pressure below the level of cerebral autoregulation due to venous pooling may result in a number of symptoms including dizziness, lightheadedness, near syncope, and ultimately syncope. Episodic syncope, defined as the complete loss consciousness and postural tone with spontaneous recovery, may be quite debilitating in individuals with postural tachycardia. Fortunately, the majority of individuals with postural tachycardia syndrome do not suffer frank syncope; however, the degree of orthostatic intolerance may be so severe as to limit daily function and contribute to a decrease in quality of life. POTS is not a single condition, but rather a heterogeneous group of conditions/disorders that have similar clinical physiological presentations.^[5,6]^ Selective serotonin reuptake inhibitors (SSRIs) are a commonly prescribed treatment for POTS patients.^[7,8]^ SSRIs increase nerve communication and stimulation of the standing vasoconstriction reflex.^[9,10]^ This reduces venous blood pooling and increases orthostatic tolerance which is beneficial in controlling the symptoms in patients with POTS.

The platelet (PL) is the major storage pool of serotonin (5HT) in the circulation containing 99% of all 5HT outside of the central nervous system; the level of circulating 5HT is a key factor for normal cardiovascular function and low levels of 5HT affect vascular tone and subsequently result in cardiac dysfunction.^[11]^ The PL does not synthesize 5HT; it is synthesized by enterochromaffin cells in the gastrointestinal tract and is bound and transported by a specific 5HT transporter (SERT) that leads to packaging of the vasoactive substance in PL dense granules.^[12,13]^ SERT has been reported in many studies to regulate the plasma levels of 5HT and its vasoconstrictive properties are well documented to regulate blood pressure.^[12]^

Insufficient peripheral 5HT may create conditions that lead to hypotension and excessive bleeding in an individual. Thus, individuals prone to syncope may be deficient in PL dense granules and 5HT. The purpose of this study was to evaluate PL dense (delta) granule storage pool deficiency (δ-SPD) in individuals diagnosed with POTS who also presented with significant bleeding history including symptoms of easy bruising, frequent nose bleeds, and for women, heavy menstrual bleeding. We hypothesized that POTS patients exhibiting clinical symptoms of mucocutaneous bleeding would be PL dense granule deficient. This is the first report of PL storage pool deficiency in a select group of patients having POTS and clinical symptoms suggestive of a PL disorder.

## 2 Methods

This study protocol conforms to the ethical guidelines of the 1975 Declaration of Helsinki as reflected in a priori approval by the Institutional Review Board of The University of Toledo Medical Center. A total of 181 patients were the subjects of this retrospective case–control evaluation. Patients included in this study had been diagnosed with POTS in our cardiology clinic via history, physical, and tilt-table test in the fasting state and also had a history of prominent bleeding symptoms for which blood had been analyzed for δ-SPD during a 10-year period (2006–2015). All had a 6 months or greater history of orthostatic intolerance manifested by orthostatic tachycardia with a heart rate increase of at least 30 BPM (or a rate exceeding 120 BPM) observed during the first 10 minutes of upright posture without orthostatic hypotension, weakness, light-headedness, fatigue, and near syncope.^[3,5]^ Although many of our patients reported frequent epistaxis, we did not consider a comorbidity of hereditary hemorrhagic telangiectasis (HHT) disease (Rendu–Osler–Weber syndrome), as notation of telangiectasis, arteriovenous malformations, nor a family history of HHT was included in their medical records.^[14]^

Tilt-table testing utilized a 70° baseline upright tilt for 30 minutes, during which time heart rate and blood pressure were monitored continually.^[5]^ The test was ended if symptomatic hypotension and bradycardia occurred, reproducing the patient's symptoms. If the patient did not experience symptoms, an intravenous infusion of isoproterenol was initiated in the supine position with a dose sufficient to raise the heart rate to 20% to 25% above the resting value. Subsequently, up-right tilt was repeated for 15 minutes. Patients who were on chronic antihypertensive, diuretic, anticholinergic, or antidepressant medications, those with diabetic neuropathy or multisystem disease of any etiology were excluded. Each patient had undergone a thorough history and physical examination as well as detailed blood chemistry analysis and thyroid profile analysis. Patients were also excluded if taking an SSRI for therapeutic management of their condition. POTS patients without a history or symptoms of bleeding were not included in the study.

Our retrospective study employed female subjects recruited to establish normal controls. Control subjects (41) were vetted using a bleeding questionnaire, aggregation assay, PL 5HT levels, and PL delta granule (DG) content determination. They were volunteers without any history of syncope episodes, without history of common bleeding symptoms and had normal numbers of PL DGs (4–6 DG/PL) and normal 5HT concentrations (200–800 ng/10^9^ PLs) extracted from PLs.

Platelet-rich plasma (PRP) was obtained from whole blood by centrifugation at room temperature for 15 minutes at 200*g*. Electron microscopy coated copper grids used for PL support were washed with deionized water following PRP incubation and air-dried. A FEI Tecnai G2 Spirit BioTwin transmission electron microscope (Hillsboro, OR) was used determine an average number of DG/PL. Previous studies from this laboratory have established a normal of 4.60 + 0.47; mean ± 1 standard deviation (SD) DG/PL, consistent with the established literature. PL 5HT concentration was determined by ELISA using commercially available kits and expressed in nanograms × 10^9^ PLs.^[15]^

Unless otherwise stated, data are presented as mean + 1 SD. Chi-square and Fisher exact test were performed using the SAS System (SAS Institute, Inc., Cary, NC) and graphed using SigmaPlot (SSPS, Inc., Chicago, IL).

## 3 Results

### 3.1 Demographics

**Figure 1:**
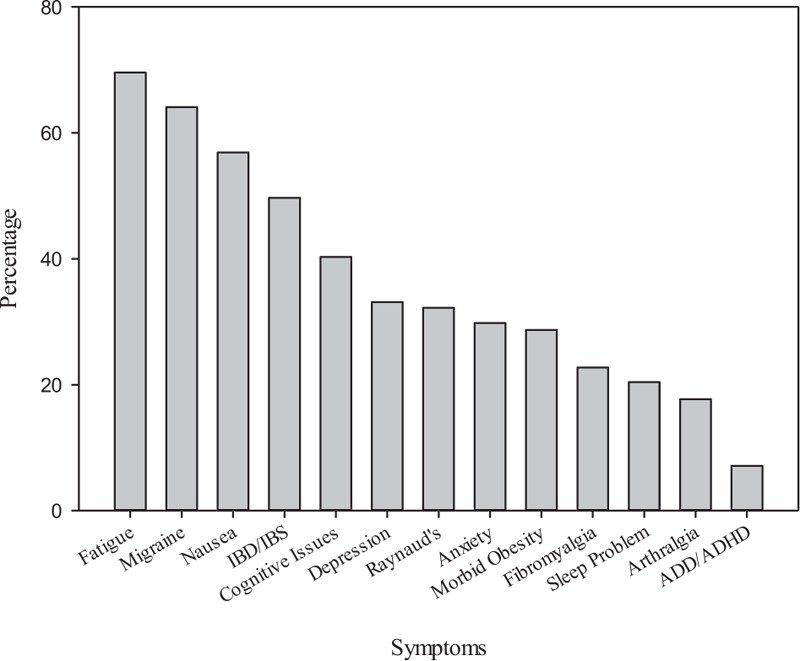
Clinical symptoms potentially related to low serotonin levels reported by POTS patients. A significant number of clinical symptoms identified with our patients have been reported to be related to low serotonin levels.

All patients included in the study were diagnosed with POTS by tilt-table analysis and all had symptoms of a bleeding diathesis including histories of frequent epistaxis, easy bruising, and/or heavy menstrual bleeding. Patient demographics are listed in Table 1. This table lists the percentage of a variety of clinical symptoms observed; some symptoms were not noted in all patient medical records, therefore, some of the calculated percentages were based upon fewer observations. Disorders associated with low 5HT levels are illustrated in Fig. 1. While we cannot be certain that these symptoms are absolutely related to diminished 5HT stores in the PLs, the constellation of clinical presentations we observed demonstrates a significant heterogeneity of symptoms suggested to be due to low 5HT as described in the literature.^[9,16,17]^

**Table 1:**
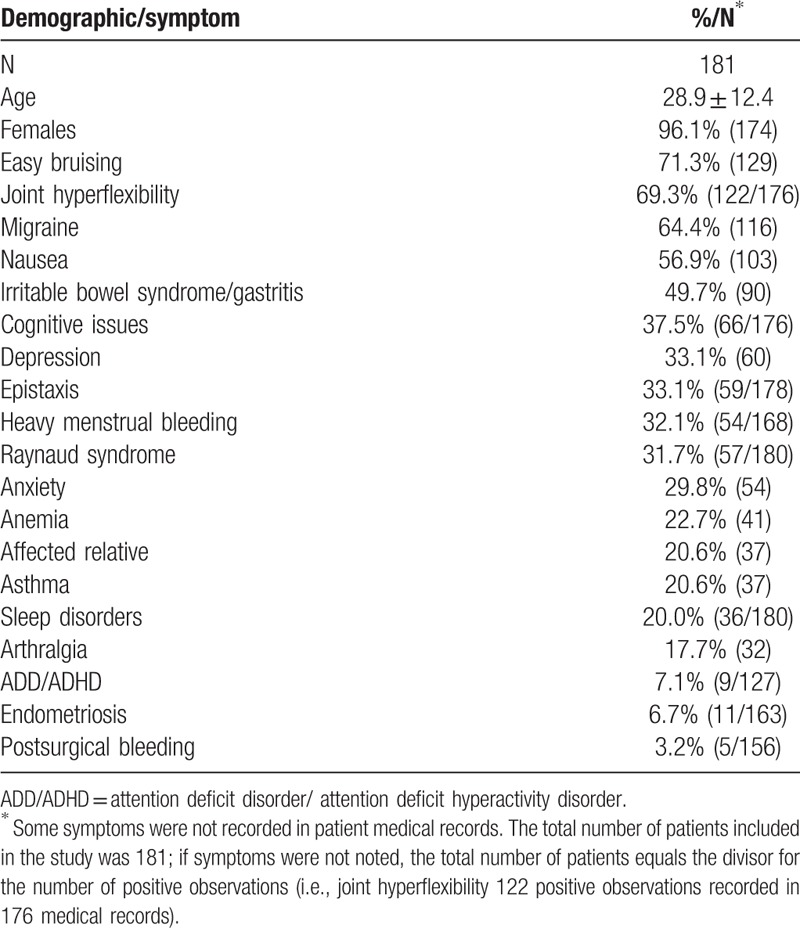
Baseline characteristics and comorbidities in patients with postural orthostatic tachycardia syndrome.

### 3.2 Joint mobility

The incidence of excessive joint mobility was significant for 69.3% of the patients included in this study with 21.6% of these having a Beighton score of 6 or greater (a score 4 or more of 8 joints plus the trunk affected (>4/9) is consistent with a diagnosis of joint hypermobility) by physical examination (*P* < 0.001). Other symptoms affecting joints and the vasculature included 31.7% (57/180, *P* < 0.001) experiencing Raynaud phenomenon and 17.7% (32/181, *P* < 0.001) complained of arthralgia.

### 3.3 Bleeding symptoms

Bleeding symptoms included easy bruising (71.3%; 129/181, *P* < 0.001), epistaxis (33.1%; 59/178, *P* < 0.001), heavy menstrual bleeding (32.1%; 54/168, *P* < 0.001), anemia (22.7%; 41/181, *P* < 0.001), endometriosis (6.7%; 11/163, *P* = 0.11), or a significant postsurgical bleeding event (3.2%; 5/156, *P* = 0.28). Patients had fewer red cells than normal as well as low hemoglobin and hematocrit consistent (all, *P* > 0.001) with the frequency of anemia described earlier (Table 2). All other cell counts were within normal limits.

**Table 2:**
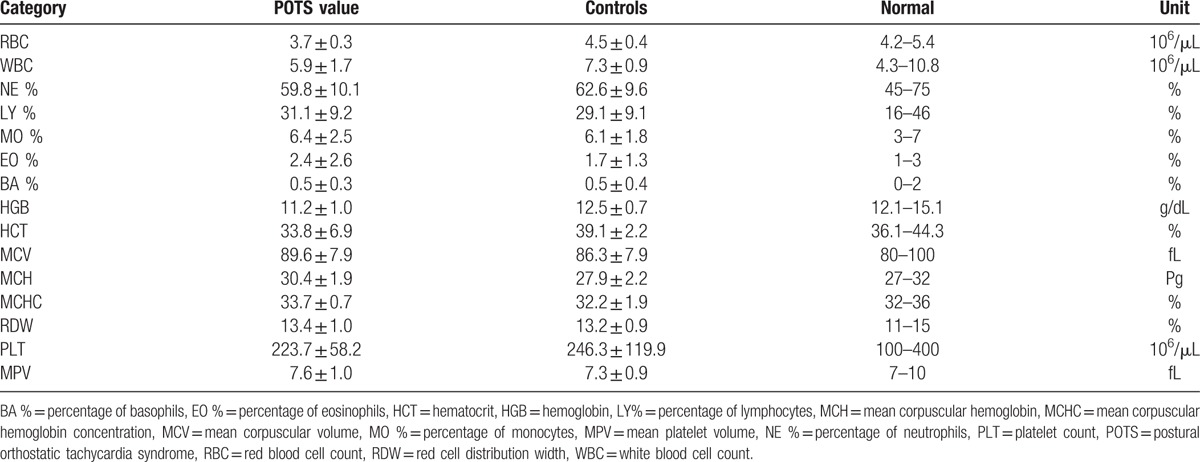
Complete blood cell count of patients diagnosed with postural orthostatic tachycardia syndrome and control subjects.

### 3.4 Platelet dense granules

**Figure 2:**
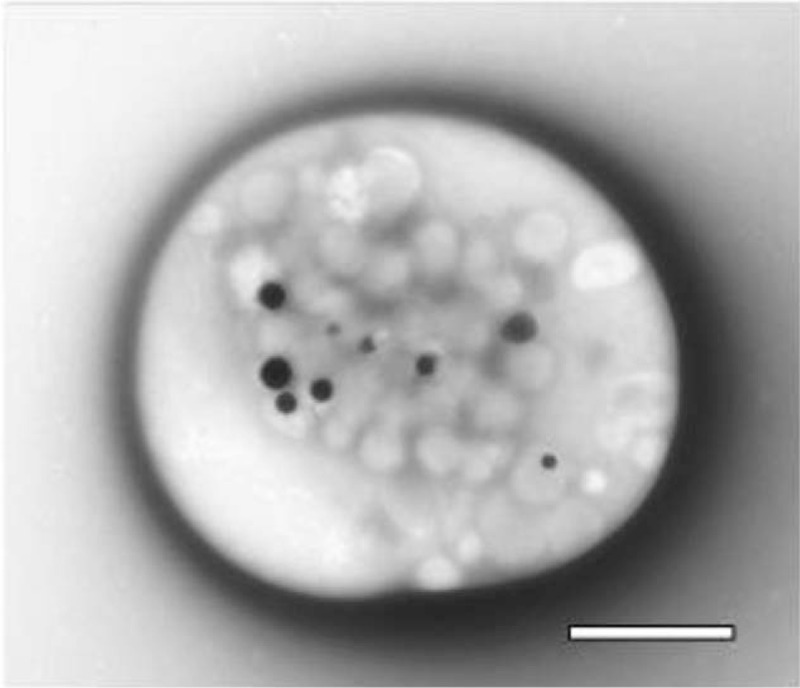
Whole-mounted platelet. This image is a whole platelet obtained from platelet-rich plasma that had settled upon an electron microscopy support film, washed and air dried without fixation. Dense granules are readily apparent due to the opacity of calcium, one of the constituents stored within delta granules. Bar = 1 μm. Original magnification: 10,000×.

**Figure 3:**
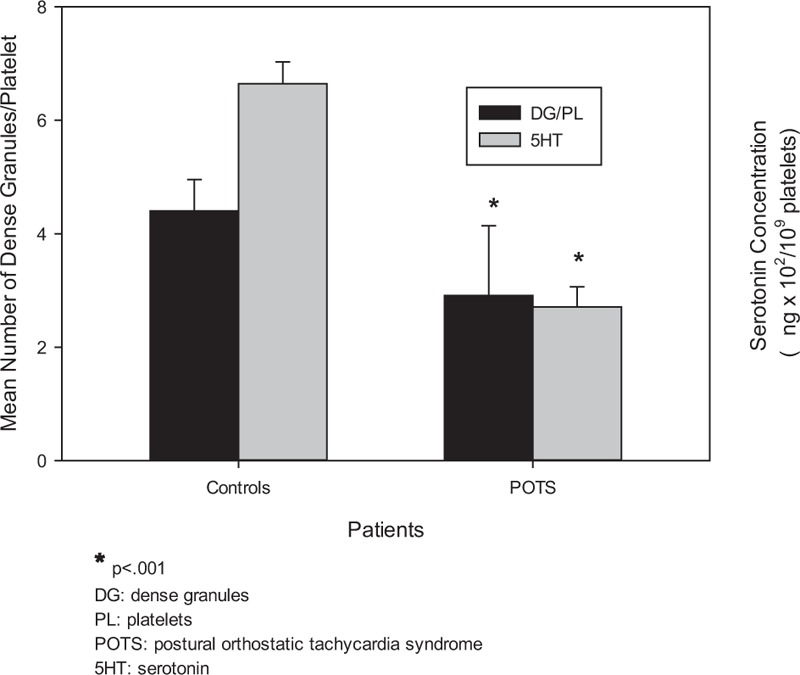
Comparison of the mean number of dense granules and of serotonin concentration extracted from platelets in control subjects and POTS patients. The mean number of platelet dense granules and serotonin levels are significantly less in POTS patients when compared with control subjects (∗*P* < 0.001).

Our patients’ average number of DG/PL was 2.91 ± 1.23 compared with our control group (4.40 ± 0.55 DG/PL, *P* < 0.001; Figs. 2 and 3). Eighty-one percent (146/181, *P* < 0.001) had a deficiency of PL dense granules.

### 3.5 Serotonin

Determination of PL 5HT concentration was added during the latter years of our study, once frequent clinical symptoms associated with low 5HT levels was recognized. 5HT extracted from PLs obtained from PRP and normalized to 10^9^ PL/mL was analyzed by ELISA for 65 of our patients. The mean 5HT concentration was 271.05 ± 35.19 ng/10^9^ PLs (±SE, *P* < 0.001; normal range 200–800 ng/10^9^ PLs). Our control group 5HT concentration was 664.15 ± 38.6 ng/10^9^ PLs (Fig. 3).

### 3.6 Other common symptoms

A variety of additional symptoms for these patients was also noted in their medical history (Table 1). Most frequently reported was cognitive issues (37.5%; 66/176, *P* < 0.001), depression (33.1%; 60/181, *P* < 0.001), anxiety (29.8%; 54/181), and sleep disorders (20%; 36/180, *P* < 0.001); attention deficit disorders were noted for 7.1% (9/127) of the patients.

## 4 Discussion

The aim of this retrospective study was to investigate a potential relationship between POTS and PL dysfunction. The study was restricted to POTS patients with anemia, and/or had clinical symptoms of bleeding, and had been evaluated by electron microscopy. The most common bleeding symptom was easy bruising (71.3%, *P* < 0.001), but frequent nose bleeds, heavy menstrual bleeding and a family history of bleeding were also frequently reported. Most of these patients had excessive joint mobility and complained of fatigue; 81% (*P* < 0.001) were diagnosed with a PL δ-SPD. Significantly lower levels of 5HT were found in POTS patients when compared to control subjects. Our data demonstrate POTS patients with bleeding symptoms have diminished PL 5HT stores supporting the hypothesis that POTS is a low 5HT disorder.^[9]^

POTS has been described as the most common form of orthostatic intolerance, affecting approximately 500,000 people in the United States.^[18]^ The diagnosis of POTS can be readily made by tilt-table assessment finding a pulse greater than 120 beats/minute or an elevation of pulse of 30 BPM in the first 10 minutes of elevation into an upright position, which is different than the fall of blood pressure as seen in vasovagal syncope under the same conditions. It is the least severe of the dysautonomias, however, dysfunction of the autonomic nervous system in POTS tends to be more severe with numerous associated symptoms than observed in typical cases of neurocardiogenic syncope.^[19,20]^

The disorder is often misdiagnosed as chronic anxiety or a panic disorder because the autonomic failure in these patients is not severe and the variety of clinical symptoms including fatigue, lightheadedness, nausea, headache, near syncope, and exercise intolerance is subtle.^[19,20]^ Furthermore, POTS may be idiopathic and unrelated to other diseases, with most affected patients categorized with a partial dysautonomic condition appearing to be related to mild peripheral autonomic neuropathy.^[19,20]^

The female/male ratio of 17:1 in our study is significantly greater than usually described at 5:1; a recent epidemiology study of 3002 patients reported a female to male ration of 3:1 with a median age of 23, a significantly younger age than other forms of syncope.^[21]^ The increased frequency of affected females in our study might be related to the specific comorbidity of bleeding diathesis; patients without bleeding symptoms had not been evaluated for δ-SPD, a limitation of our study. With the heterogeneity of symptoms associated with POTS, a variety of proposed etiologies have been suggested yet no conclusive basis nor mechanism have been established.^[22–26]^

We report a significant relationship with PL δ-SPD that may explain many of the symptoms reported with POTS. Patients previously diagnosed with POTS by tilt-table assessment with additional prevalent bleeding symptoms were also evaluated for δ-SPD by electron microscopy. For the entire group of 181 POTS patients, the mean of 2.91 ± 1.23 DG/PL is significantly different than control subjects (4.40 ± 0.55 DG/PL (*P* < 0.001)); 81% (147/181) were diagnosed with δ-SPD. PL dysfunction disorders usually manifest with common symptoms including easy bruising, frequent nose bleeds, heavy menstrual bleeding for women, excessive bleeding of the gums with brushing, and excessive bleeding with surgery or trauma. The condition is thought to have a dominant inheritance pattern but is also known to be acquired. Interestingly, 1 drug that has been shown to reduce bleeding symptoms related to δ-SPD (desmopressin) has also been reported to reduce symptoms in POTS.^[27]^

As stated previously, only patients diagnosed with POTS with a bleeding history, symptoms of bleeding, and/or anemia were included in this study. PL 5HT concentration was not determined during many of the early years when patients were initially evaluated for PL dysfunction, however our 5HT data are sufficient (n = 65) to conclude PL 5HT concentration was significantly decreased in patients with POTS compared with control subjects (*P* < 0.001).

The diminished concentration of 5HT in our patients appears to trend with decreased numbers of PL dense granules; 5HT is stored in these organelles. The variety of complaints such as fatigue, migraine headache, nausea, and irritable bowel syndrome/disease has been reported due to low 5HT. We found no statistical evidence of correlation via a number of analyses for symptom associations. Patients without bleeding symptoms but having symptoms related to low 5HT would suggest a causal, rather than correlative, relationship with PL dysfunction. Conversely, PL dysfunction and bleeding could be unrelated to POTS.

The PL dense granules contain ADP, ATP, 5HT, calcium, and pyrophosphates at different concentrations (e.g., there is 9–10 times as much ADP than ATP in dense granules with the converse of adenine nucleotide concentrations in the PL cytosol). The transporters for PL uptake of dense granule constituents are different for each of these substances; it is not unreasonable to assume that there could be a PL storage pool deficiency for any of these substances independent of PL number and function. A defect of SERT might lead to low levels of 5HT within PL dense granules but would not affect dense granule number nor ADP concentration. Studies have defined δ-SPD as a condition of increased PL ATP/ADP ratios exceeding 2.0 directly related to diminished granular ADP stores and that decreased ADP levels correlate with reduced numbers of PL dense granules.^[28–30]^ We and others have found that δ-SPD and diminished PL 5HT stores also correlate with one another.^[31,32]^ Although we have identified a significant deficiency of the PL dense granules in these patients, not all of the patients were diagnosed with δ-SPD; 19% were found to be within normal limits.

## 5 Conclusions

We report an association of PL δ-SPD, with low PL concentrations of 5HT in POTS patients who also exhibited symptoms of a potential bleeding diathesis. A number of comorbidities in these patients may be explained by having low 5HT levels in the circulation. These results suggest a potential role of diminished synthesis of 5HT or a potential PL dysfunction to consider for study for the etiology of POTS. Future studies planned include a case–control study that will utilize a formal bleeding checklist as used for the establishment of our control group, assessment of menstrual bleeding using a vetted scoring rubric, and inclusion of all primary POTS patients with and without bleeding symptoms to further evaluate our postulates.

## Acknowledgment

We are indebted to Dr Sadik Khuder, Professor of Medicine at the University of Toledo and an expert statistician for review of our data.
